# Facile aqueous synthesis of hollow dual plasmonic hetero-nanostructures with tunable optical responses through nanoscale Kirkendall effects†

**DOI:** 10.1039/d2na00606e

**Published:** 2022-11-10

**Authors:** Mariia Ivanchenko, Alison L. Carroll, Andrea B. Brothers, Hao Jing

**Affiliations:** Department of Chemistry and Biochemistry, George Mason University Fairfax Virginia 22030 USA hjing2@gmu.edu; Department of Chemistry, American University Washington DC 20016 USA

## Abstract

Herein, we report the colloidal synthesis of hollow dual-plasmonic nanoparticles (NPs) using Au@Cu_2_O core–shell NPs as templates and exploiting the nanoscale Kirkendall effect. In our synthesis, we used organic compounds as a source of chalcogenide ions for an anion exchange reaction at elevated temperatures using polyvinylpyrrolidone (PVP) as a capping reagent to transform the solid Cu_2_O shell into a hollow copper chalcogenide shell. The resulting structures possess different features depending on the chalcogenide precursor employed. TEM images confirm the complete transformation of Au@Cu_2_O templates when 1,1-dimethyl-2-selenourea was added and the formation of hollow Au@Cu_2−*x*_Se nanostructures. In contrast, residues of Cu_2_O attached to the Au core were present when thioacetamide was used for the synthesis of Au@Cu_2−*x*_S with all other conditions kept the same. The divergence of architectures caused distinct optical properties of Au@Cu_2−*x*_S and Au@Cu_2−*x*_Se NPs. This synthetic approach is an effective pathway for maneuvering the size of interior voids by varying the concentration of chalcogenide ions in the reaction mixture. The insights gained from this work will enrich the synthetic toolbox at the nanoscale and guide us on the rational design of multicomponent plasmonic nanoparticles with precisely controlled hollow interiors and sophisticated geometries, further enhancing our capabilities to fine-tune the electronic, optical, compositional, and physicochemical properties.

## Abstract 2

Hollow dual-plasmonic Au@Cu_2−*x*_Se and Au@Cu_2−*x*_S NPs have been synthesized through a wet chemistry route, involving template formation, an anion exchange, and the Kirkendall effect, and demonstrated different optical and structural features.

## Graphical abstract

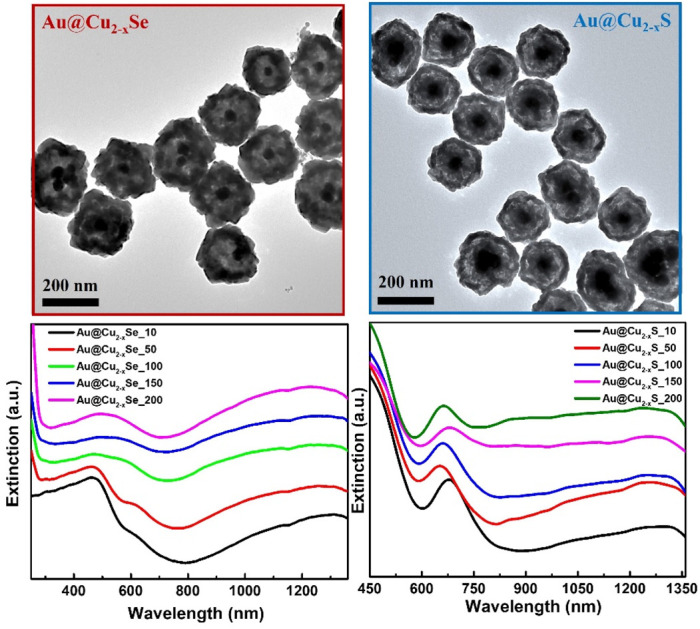

## Introduction

Multicomponent plasmonic nanoparticles, especially structurally well-defined noble metal-nonstoichiometric copper chalcogenide (p-type) dual plasmonic hybrid hetero-nanostructures, have emerged as an intriguing platform of superstructures due to their synergistically reinforced optical properties.^1,2^ The integration of two intrinsically dissimilar plasmonic constituents in one nano-entity empowers us to explore many new-yet-unexpected features, holding tremendous potential for plasmon-mediated energy transfer, plasmon-enhanced photocatalysis, and plasmon-related photothermal and photodynamic theranostics. Over the past few decades, research on dual plasmonic nanostructures has been burgeoning^3–9^ and much progress has been made with respect to the synthesis of a wide variety of dual plasmonic nanostructures with distinctive material compositions, sizes, or shapes.^10–19^ Among a plethora of dual plasmonic hetero-nanostructures obtained, hollow nanocrystals comprising noble metal cores and nonstoichiometric copper chalcogenide shells are particularly intriguing owing to their unique properties such as large surface areas, high pore volumes, reduced charge recombination, low densities, and accelerated mass transfer dynamics.^20,21^ As is known, hollow nanostructures are interesting functional nanomaterials for a variety of applications such as catalysis,^22,23^ energy storage,^24^ lithium-ion batteries,^25^ drug delivery,^26,27^ and biomedicine.^7,28,29^ It is also anticipated that cavities in hollow nanostructures of dual plasmonic nanostructures will be able to promote superior plasmonic properties than their solid counterparts, in which the core is coated with a dense shell, due to the plasmon hybridization mechanism that results in the enhancement of the electromagnetic field.^30,31^ Therefore, engineering dual plasmonic hybrid nano-systems with hollow interiors based on a Au NP core in a nonstoichiometric copper chalcogenide hollow shell is important, not only for a purely fundamental study, but also for practical applications of such complex multifunctional nanomaterials in technology.

A variety of methods have been elaborated to produce hollow dual plasmonic nanostructures, such as soft or hard template approaches, galvanic replacement reaction, chemical etching, the Kirkendall effect, and Ostwald ripening.^32^ Among them, the hard template method based on Au@Cu_2_O core@shell nanoparticles (NPs) is the most effective in the production of hollow dual-plasmonic Au@Cu_2−*x*_S nanostructures.^2^ The process of interior void formation in a solid shell and its conversion from Cu_2_O into Cu_2−*x*_S takes place through anion exchange with the nanoscale Kirkendall effect.^33^ The hollow structure is produced as a result of the discrepancy in anion diffusion rates.^34^ In the course of the reaction, oxygen ions in the shell of the template NPs are extracted and replaced through the addition of sulfide ions. Because the diffusion of species outward is much quicker than the inward diffusion of chalcogenide anions, small vacancies are formed inside the template shell that coalesce into a large single void converting the core@shell template into a hollow Au@Cu_2−*x*_S nanostructure.^35–37^ This approach permits the tunability of the size and shape of the Au@Cu_2−*x*_S hollow nanostructures, changing their chemical and physical properties.^7^ The as-obtained hollow Cu_2−*x*_S shell maintains the original shape of the templates.^38,39^ This method can be used to fabricate even more complex nanostructures with multiple hollow Cu_2−*x*_S shells surrounding the core.^40^ However, studies on the compositional and structural diversity of such hollow dual plasmonic nanoparticles are still largely underdeveloped since all reports published so far are only focused on Au@Cu_2−*x*_S.^7,29,41–43^ To the best of our knowledge, synthesis and characterization of hollow dual plasmonic Au@Cu_2−*x*_Se hybrid nanostructures have not been reported yet, besides very scarce synthetic protocols published for bare hollow copper selenide nanoparticles.^44,45^ In light of the important applications of selenium-containing nanoparticles nowadays in energy storage^46,47^ and cancer treatment,^48,49^ it is fundamentally and technologically imperative to develop a deeper understanding of the design and synthesis of such nanostructures with deliberately controlled morphologies, compositions, and surface architectures.

In this study, hollow dual-plasmonic Au@Cu_2−*x*_Se hetero-nanostructures with geometrically tunable localized surface plasmon resonances (LSPRs) were prepared for the first time in aqueous solution through a robust and facile synthetic approach mediated by the nanoscale Kirkendall effect. We used colloidal Au@Cu_2_O core–shell nanoparticles as the sacrificial template for the hollowing process. The chemical transformations of pre-formed sacrificial templates to hollow structures were performed using a water-soluble organic molecule, 1,1-dimethyl-2-selenourea [(CH_3_)_2_NC(Se)NH_2_], as the source of Se^2−^ anions at an elevated temperature. Besides Se^2−^ ions, another hydrophilic organic molecule, thioacetamide [CH_3_C(S)NH_2_], was used as the source of S^2−^ anions to generate hollow nano-architectures with identical conditions in order to better understand and compare the Kirkendall effects on the structural and morphological evolutions of nanoscale materials in aqueous solutions. Two drastically distinctive structural evolution processes were observed on dual-plasmonic Au@Cu_2−*x*_S and Au@Cu_2−*x*_Se hybrids with hollow interiors. The different interior hollowing behaviors, driven by the nanoscale Kirkendall effect in Au@Cu_2−*x*_S and Au@Cu_2−*x*_Se hybrids, resulted in distinctive yet tunable optical responses dictated by two dissimilar LSPRs originating from the collective oscillations of free electrons and free holes in the metal and semiconductor, respectively. While the Cu_2_O shell in the presence of a Se precursor was entirely converted into Cu_2−*x*_Se, some residual Cu_2_O was present after the reaction with the S precursor under the same conditions. This is also the first time, hollow dual plasmonic nanostructures, synthesized with two distinctive organic chalcogenide precursors through the nanoscale Kirkendall effect, were systematically compared in terms of their increased architectural complexities and tuned optical characteristics.

## Experimental

### Chemicals and materials

Hydrogen tetrachloroaurate(iii) trihydrate (HAuCl_4_·*x*H_2_O, 99.995%), potassium carbonate (K_2_CO_3_, 99.995%), formaldehyde (37 wt% solution in water), polyvinylpyrrolidone (PVP, average MW 55 000), ethanol (C_2_H_5_OH, 95 vol%), copper(ii) nitrate hydrate (Cu(NO_3_)_2_·H_2_O, 99.999%), sodium hydroxide (NaOH, ≥98%), hydrazine (N_2_H_4_·3H_2_O, 35 wt% solution in water), thioacetamide (CH_3_C(S)NH_2_, ≥99.0%), 1,1-dimethyl-2-selenourea ((CH_3_)_2_NC(Se)NH_2_, 97%). All the chemicals used for the synthesis were of analytical grade and were used without further purification. All solutions were prepared using deionized water (18.2 MΩ cm resistivity).

### Synthesis of quasi-spherical Au nanoparticles

50 mg of K_2_CO_3_ was dissolved in 200 mL of water. Then, 3 mL of 25 mM HAuCl_4_ was added. After the mixture was aged in the dark overnight, 1.334 mL of formaldehyde solution was added to the mixture under vigorous magnetic stirring (500 rpm). A brick-red colloidal suspension began to form after ∼5 min. The colloidal solution was kept under stirring for 30 min. Then, Au nanoparticles were collected by centrifugation at 2000 rcf for 10 minutes and washed with 20 g per L polyvinylpyrrolidone aqueous solution and ethanol in sequence and finally redispersed in 10 mL of water.

### Synthesis of Au@Cu_2_O core–shell nanoparticles

100 μL of colloidal Au quasi-spherical nanoparticles was first introduced into 5 mL of 20 g per L PVP aqueous solution. 10 μL of 0.1 M Cu(NO_3_)_2_ solution was subsequently added while stirring the solution at 700 rpm. Then, 11.2 μL of 5 M NaOH and 5 μL of N_2_H_4_ solution were added. The solution changed color from purple to blue and then to green. The solution was kept under stirring for 30 min and the resulting core–shell nanoparticles were subsequently washed with water by centrifugation (5000 rpm for 5 min) and redispersed in 2 mL of water.

### Synthesis of Au@Cu_2−*x*_S hollow nanospheres

Various volumes (10, 50, 100, 150, and 200 μL) of freshly prepared 50 mM thioacetamide solution were added to as prepared Au@Cu_2_O and reacted at 75 °C for 2 h. The color change of the solution to light brown was observed. The as-prepared hollow nanospheres were centrifuged at 4500 rpm for 5 min and washed with water.

### Synthesis of Au@Cu_2−*x*_Se hollow nanospheres

Various volumes (10, 50, 100, 150, and 200 μL) of freshly prepared 50 mM 1,1-dimethyl-2-selenourea aqueous solution were added to as prepared Au@Cu_2_O and reacted at 75 °C for 2 h. The color of the solutions varied from brown to black (from a lower to higher concentration of Se-precursor added). The as-prepared hollow nanospheres were centrifuged at 4500 rpm for 5 min and washed with water.

### Methods and instrumentation

Optical extinction measurements were performed using a Shimadzu UV-2600 Plus spectrophotometer equipped with quartz cuvettes of 0.5 cm optical path length. Transmission electron micrographs (TEM) were recorded using a JEOL JEM-1400Flash microscope operating at 120 kV. High-resolution transmission electron microscopy (HRTEM) images were collected utilizing a JEM 2100Plus TEM operating at 200 kV. The colloidal solutions of nanoparticles were coated onto a 300 mesh copper grid that was dried completely at room temperature before the measurements. Energy dispersive X-ray spectroscopy (EDS) measurements were conducted on a SEM equipped with an EDAX APEX detector. Elemental mapping was performed on a JEM 2100Plus TEM with a JED-2300T EDS detector. Samples were deposited on a Ni grid to avoid the interference of peaks from the Cu grid and fixed on a JEOL single tilt beryllium retainer for EDS analysis. The surface morphology of the NPs was analyzed using a JEOL JSM-IT500HR scanning electron microscope (SEM). Powder X-ray diffraction (XRD) patterns of the samples were obtained at room temperature for 2 h with the scattering angle 2*θ* range from 25° to 70°, using a benchtop Miniflex-600 powder X-ray diffractometer (Cu K_α_, *λ* = 1.5418 Å).

## Results and discussion

**Fig. 1:**
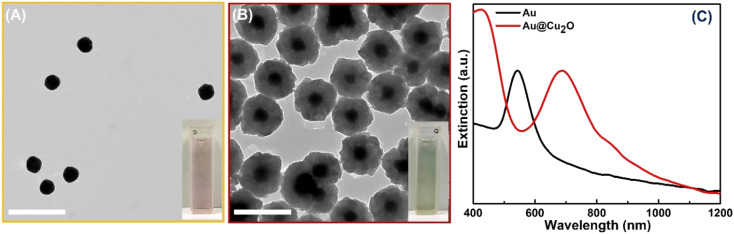
Formation of the sacrificial hard templates. TEM images of (A) Au quasi-spherical nanoparticles and (B) Au@Cu_2_O core–shell NPs. Insets: color pictures of corresponding colloidal solutions of Au quasi-spherical NPs and Au@Cu_2_O core–shell NPs. Scale bars correspond to 200 nm. (C) The extinction spectra of colloidal Au and Au@Cu_2_O NPs (cuvette path length is 0.5 cm).

The synthesis of hollow dual plasmonic NPs is a three-step process based on the hard template method. Firstly, Au quasi-spherical cores were fabricated with an average diameter of ∼50 nm and stabilized in PVP (Fig. 1A). The Au NP suspension appeared light pink in color and the corresponding extinction peak was centered at 545 nm (Fig. 1C). Secondly, a Cu_2_O shell with a thickness of ∼45 nm was coated on Au NPs to serve as a sacrificial template in the next step. Formed multifaceted Au@Cu_2_O core–shell nanoparticles were visible in the TEM image (Fig. 1B). Deposition of the shell induced a red shift of the extinction peak to 685 nm due to the higher refractive index of Au NPs surroundings (Fig. 1C). As a result, Au@Cu_2_O colloidal solution appeared green in color. The uniformity of the semiconducting shell was further evidenced by the distribution of the Cu_2_O shell thickness shown in Fig. S1.† As can be observed in the HRTEM images, the shells were epitaxially grown on the surface of the gold NPs because of the minimal mismatch between Au and Cu_2_O cubic crystal structures (Fig. S2A†). The orientation of the crystalline Cu_2_O shell is driven by the Au core because atoms of the growing material are directed to the surface in a way to minimize its energy during hetero-nucleation. Therefore, the (111) planes of Au are aligned in the same directions as the (111) planes of Cu_2_O, and lattice parameters were measured to be 0.236 nm and 0.246 nm, respectively, which agree with the results reported previously (Fig. S2B†).^50^

**Fig. 2:**
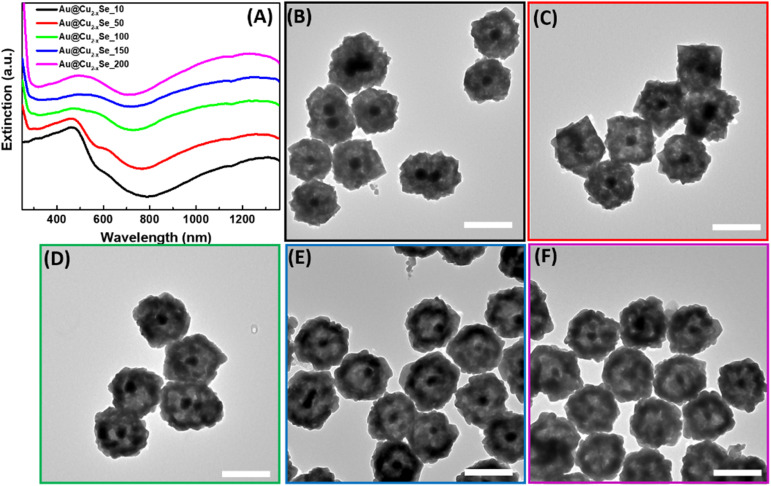
Characterization of hollow Au@Cu_2−*x*_Se NPs. (A) UV-vis-NIR extinction spectra of aqueous solutions of hollow Au@Cu_2−*x*_Se NPs obtained using 10, 50, 100, 150, and 200 μL of 50 mM (CH_3_)_2_NC(Se)NH_2_ for synthesis. TEM images of hollow Au@Cu_2−*x*_Se nanostructures obtained using (B) 10 μL, (C) 50 μL, (D) 100 μL, (E) 150 μL, and (F) 200 μL of 50 mM (CH_3_)_2_NC(Se)NH_2_ aqueous solution. Scale bars correspond to 200 nm.

The third step is a transformation of the shell through a Kirkendall process to substitute oxygen anions with chalcogenide and produce hollow structures. The formation of dual plasmonic Au@Cu_2−*x*_Se was confirmed by the appearance of two peaks in the visible and near infra-red (NIR) part of extinction spectra, corresponding to the LSPR of Au and Cu_2−*x*_Se, respectively (Fig. 2A). The strong light absorption at wavelengths below 500 nm in the extinction spectra was due to the occurrence of the interband electronic transitions from the valence band (VB) to the conduction band (CB) of the semiconductor Cu_2−*x*_Se. The addition of the Se-precursor at elevated temperatures enabled the conversion of Au@Cu_2_O into the hollow Au@Cu_2−*x*_Se nanostructures because of the thermal decomposition of 1,1-dimethyl-2-selenourea, which ensured a sufficient amount of selenide in the reaction mixture, as well as an increase of Se, Cu, and O ions diffusion rates. During the anion-exchange reaction, the Se^2−^ reacted with the Cu^+^ in the Cu_2_O shell and caused the outward diffusion of O^2−^. As Se^2−^ anions continuously approached the copper oxide, the Cu_2−*x*_Se shell thickened. The process is thermodynamically favorable, as copper chalcogenides are less soluble in polar solvents than copper oxide. Because the size of selenium anions is larger than oxygen anions, obtained hetero-nanostructures enlarged after the transformation to an average diameter of ∼200 nm. Also, it is likely that a mass diffusion of Cu_2_O clusters from the inside of heterostructures, close to the initially formed Cu_2−*x*_Se, took place because the mobility of the smaller oxygen anions is faster than that of Se^2−^. Described factors contribute to the formation of the inner pore between Au and Cu_2−*x*_Se. TEM images confirm the appearance of an interior void around the Au core (Fig. 2B–F). Also, the effect of the selenide amount added during the synthesis on the size of the void was investigated. As could be observed from the micrographs, the size of the inner pores and thickness of the Cu_2−*x*_Se shells increase as more Se-precursor is present in the reaction mixture during synthesis. This is accompanied by a slight bathochromic shift of the extinction peak corresponding to Au cores. The appearance of peaks at ∼600 nm for samples with a smaller volume of Se-precursor added could be explained by the presence of remaining Cu_2_O attached to the Au core because the concentration of Se^2−^ in the reaction mixture is not sufficient to complete the conversion to Au@Cu_2−*x*_Se. An interplanar distance of 0.333 nm was measured in the shell area of Au@Cu_2−*x*_Se NP in the HRTEM image (Fig. S3†), which agrees well with the (111) lattice planes of the Cu_2−*x*_Se crystal.^16,18^ SEM images show that hetero-nanostructures have roughened surfaces and irregular stellated icosahedral morphologies (Fig. S4†). The Se : Cu ratios for all synthesized samples were determined by energy dispersive spectroscopy. EDS analysis shows that Se : Cu atomic ratios increase as the total volume of Se-precursor added during the synthesis increases (Fig. S5†). For Au@Cu_2−*x*_Se samples fabricated using 10, 50, 100, and 200 μL of 50 mM 1,1-dimethyl-2-selenourea, *x* values were calculated to be 0.4, 0.6, 1.1, 1.4, respectively.

**Fig. 3:**
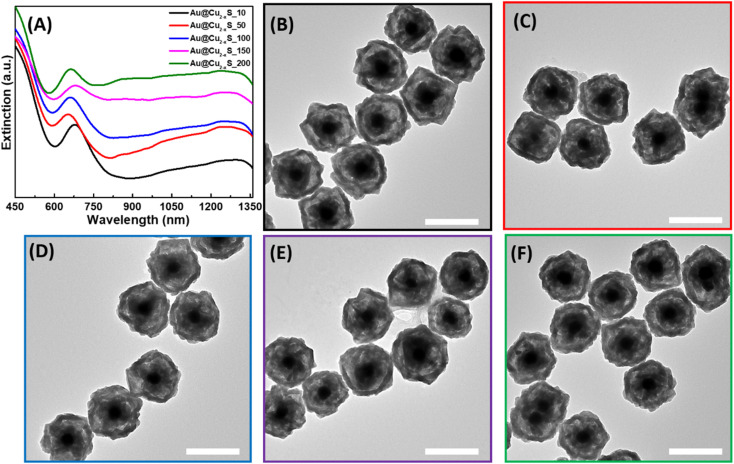
Characterization of hollow Au@Cu_2−*x*_S NPs. (A) UV-vis-NIR extinction spectra of aqueous solutions of hollow Au@Cu_2−*x*_Se NPs obtained using 10, 50, 100, 150, and 200 μL of 50 mM CH_3_C(S)NH_2_ for synthesis. TEM images of hollow Au@Cu_2−*x*_S nanostructures obtained using (B) 10 μL, (C) 50 μL, (D) 100 μL, (E) 150 μL, and (F) 200 μL of 50 mM CH_3_C(S)NH_2_ aqueous solution. Scale bars correspond to 200 nm.

Similarly, hollow Au@Cu_2−*x*_S hetero-nanostructures were synthesized using thioacetamide as an organic S-precursor. Two LSPR modes of the Au core and Cu_2−*x*_S shell were observed in extinction spectra (Fig. 3A). However, the position of the Au peak was red-shifted to ∼685 nm compared to the LSPR of Au in hollow Au@Cu_2−*x*_Se. As shown in the TEM images of hollow Au@Cu_2−*x*_S, hetero-nanostructures with only a partially converted Cu_2_O interior were formed, and the remaining Cu_2_O is still attached to the Au core. This explains the same position of the Au LSPR mode in the extinction spectra of Au@Cu_2_O and hollow Au@Cu_2−*x*_S NPs (Fig. 3). This evidences that the ion exchange takes place only on the external surface of the sacrificial templates. Simultaneously with the anion-exchange reaction, the outer layer formed a dense Cu_2−*x*_S shell due to aging processes preventing further diffusion of S^2−^ inside and blocking the transformation of the shell. The HRTEM images provide additional evidence that the outer Cu_2−*x*_S shell was formed (Fig. S6†). The lattice spacing was measured to be 0.302 nm and is consistent with the (115) planes of copper sulfide.^29,51^ SEM photographs display an irregular truncated polyhedron morphology of Au@Cu_2−*x*_S hetero-nanostructures (Fig. S7†). In SEM images at high and low magnification obtained by a secondary electron detector, the Au core inside each NP could be identified, which again indicates the formation of a very thin Cu_2−*x*_S shell. The results of EDS analysis demonstrate the increase of S-content present in hollow Au@Cu_2−*x*_S as the volume of thioacetamide added during the synthesis increased (Fig. S8†). However, EDS data cannot be used for quantitative analysis and do not represent accurate Se : Cu atomic ratios because the intensity of Cu signals reflects the total amount of Cu present in hetero-nanostructures as Cu_2_O and Cu_2−*x*_S. Therefore, *x* values for the formed Cu_2−*x*_S shells cannot be determined.

**Fig. 4:**
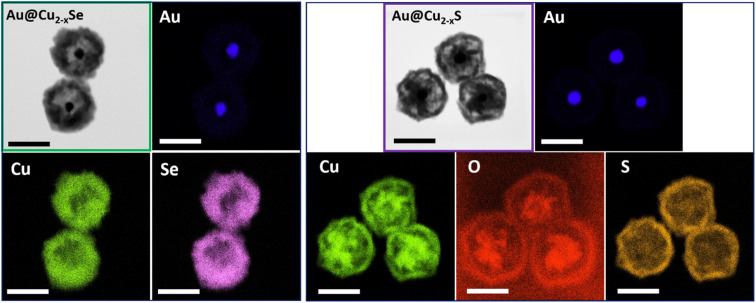
Elemental distributions of Au, Cu, and Se in hollow dual-plasmonic Au@Cu_2−*x*_Se (left panel). Mapping images of Au, Cu, O, and S in hollow dual-plasmonic Au@Cu_2−*x*_S (right panel). Scale bar: 200 nm.

The architecture divergence of the obtained dual-plasmonic nanomaterials was confirmed by EDS elemental mapping showing different elemental distributions in those nanostructures. The EDS mapping images of the Au@Cu_2−*x*_Se NPs show the distribution of Cu and Se elements mainly in the outer layers of the nanostructure, leaving the empty space of low signal intensity inside NPs and suggesting the formation of a Cu_2−*x*_Se shell. The Au element is positioned inside the hetero-nanostructures and displays a quasi-spherical inner core. In the EDS mapping images of the Au@Cu_2−*x*_S NPs, the Cu component exhibits intense signals at the edge and in the center, while the S signal mainly locates at the surface of hereto-nanostructures. On the other hand, O and Au signals mainly appeared in the center of NPs, which further confirms the presence of remaining Cu_2_O attached to the Au core (Fig. 4).

In order to obtain more crystallographic details, XRD analyses were performed for the samples obtained using various volumes of 50 mM chalcogenide precursors solutions. The characteristic XRD patterns of Au@Cu_2_O NPs and two samples of hollow dual-plasmonic Au@Cu_2−*x*_Se nanoparticles are presented in Fig. S9.† The XRD patterns confirm the phase transformation from Cu_2_O to copper selenide. The shift to higher copper deficiency for samples of hollow Au@Cu_2−*x*_Se obtained using 10 and 200 μL organic selenium precursor (CH_3_)_2_NC(Se)NH_2_, reflected in the XRD patterns of Fig. S9,† is consistent with the blue-shift of LSPRs peak in the experimentally measured extinction spectra observed in Fig. 2. Notwithstanding the slight differences in *x* values compared to those obtained from EDX, the diffraction patterns of Au@Cu_2−*x*_Se_10 μL and Au@Cu_2−*x*_Se_200 μL in the diffractogram match very well those in berzelianite Cu_1.8_Se and CuSe nanoparticles, respectively, which indicates the increasing free carrier concentrations and copper deficiencies in plasmonic non-stoichiometric copper chalcogenides.

We observed the same trend in the degree of copper deficiencies in XRD spectra for hollow dual-plasmonic Au@Cu_2−*x*_S obtained with increasing amounts of organic sulfur precursor CH_3_C(S)NH_2_ as evidenced in Fig. S10,† further substantiating the formation of hollow hybrid nanostructures comprising a Au core and non-stoichiometric copper chalcogenides shell through the Kirkendall effects. Interestingly, the patterns of the sample Au@Cu_2−*x*_S_10 μL match those in CuO nanoparticles, indicating the oxidation of the cuprous oxide (Cu_2_O) shell at the beginning of the ion-exchange process in the presence of small amounts of CH_3_C(S)NH_2_ in the solution.

## Conclusions

In conclusion, we have described a facile template synthesis of hollow dual-plasmonic Au@Cu_2−*x*_S and Au@Cu_2−*x*_Se hetero-nanostructures through an anion exchange reaction with the Kirkendall effect from Au@Cu_2_O sacrificial template NPs. The as-synthesized hollow NPs possess distinct optical properties and different structural features determined by the source of chalcogenide ions used during synthesis. Also, the effects of the chalcogenide precursor concentration used during synthesis on the void size of the produced NPs were studied. The transformation of the Au@Cu_2_O core@shell template into the hollow Au@Cu_2−*x*_Se nanostructures was complete under reaction conditions used, while Au@Cu_2−*x*_S hetero-nanostructures with a partially converted Cu_2_O interior were formed and part of the solid Cu_2_O shell remained attached to the Au core. The structural disparity of hollow Au@Cu_2−*x*_Se and Au@Cu_2−*x*_S NPs was observed in TEM images and EDS elemental maps and explained their different optical properties. Our findings show a diverse range of multifunctional hollow dual-plasmonic nanostructures because hollow dual-plasmonic Au@Cu_2−*x*_Se hetero-nanostructures were fabricated, characterized, compared with analogies Au@Cu_2−*x*_S NPs and reported for the first time. We can enthusiastically envision that this facile approach based on the nanoscale Kirkendall effect could be extended to other similar nano-systems through deliberate control over geometries and optical responses, which further stimulate the development of novel hollow dual-plasmonic nanostructures containing other chalcogens, such as tellurium (Te), particularly with the most recent advances of Te-based nanomaterials in nano-reactors and theranostic nano-agents for tumor therapy.^52^

## Author contributions

Hao Jing conceived and designed the research. M. I. and A. L. C. synthesized the hollow dual plasmonic nanoparticles and collected the extinction spectra. M. I. used scanning (JEOL JSM-IT500HR) and transmission electron microscopy (JEOL JEM-1400Flash) to characterize all nanostructures. A. B. B. obtained high-resolution and STEM elemental mapping images on all nanoparticles using a JEM 2100Plus TEM equipped with a JED-2300T EDS detector. Hao Jing supervised the research. Hao Jing and M. I. co-wrote the paper. All authors discussed the results and commented on the manuscript.

## Conflicts of interest

The authors declare no competing financial interest.

## Supplementary Material

NA-005-D2NA00606E-s001
