# Supplementary material for: Facile aqueous synthesis of hollow dual plasmonic hetero-nanostructures with tunable optical responses through nanoscale Kirkendall effects
Source: Nanoscale Adv. 2022 Nov 10;5(1):88–95. doi: 10.1039/d2na00606e (PMC9765514; doi:10.1039/d2na00606e)
Supplement: NA-005-D2NA00606E-s001 [file NA-005-D2NA00606E-s001.pdf]

Electronic Supplementary Material (ESI) for Nanoscale Advances.
This journal is © The Royal Society of Chemistry 2022

Supporting Information for

Facile Aqueous Synthesis of Hollow Dual Plasmonic Hetero-Nanostructures with Tunable Optical Responses through the Nanoscale Kirkendall Effects

Mariia Ivanchenko,[†] Alison L. Carroll,[†] Andrea B. Brothers,[§] and Hao Jing[†, *]

[†] Department of Chemistry and Biochemistry, George Mason University, Fairfax, Virginia 22030, USA

[§] Department of Chemistry, American University, Washington, DC 20016, USA

* To whom correspondence should be addressed.

Email: hjing2@gmu.edu

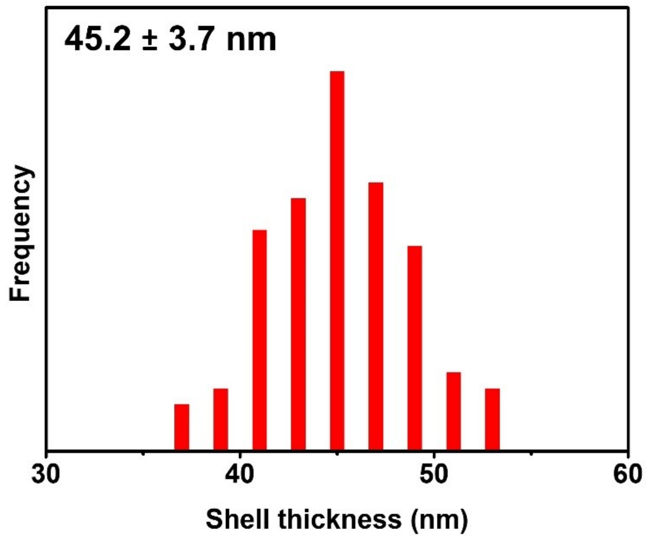

Fig. S1. Distribution of Cu_2O shell thickness obtained from TEM images of Au@Cu_2O NPs.

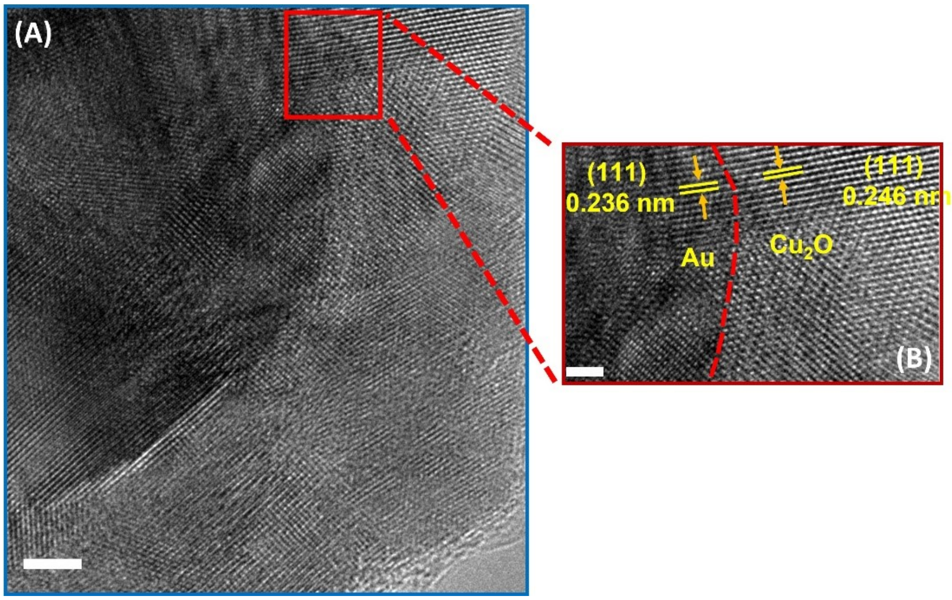

Fig. S2. (A) HRTEM image of an Au@Cu_2O nanoparticle with denoted interface line that was examined. The scale bar is 10 nm. (B) HRTEM image of an Au@Cu_2O nanoparticle with indicated interplanar spacings corresponding to the lattice fringes of the (111) planes of the Au core and Cu_2O shell. The scale bar is 5 nm.

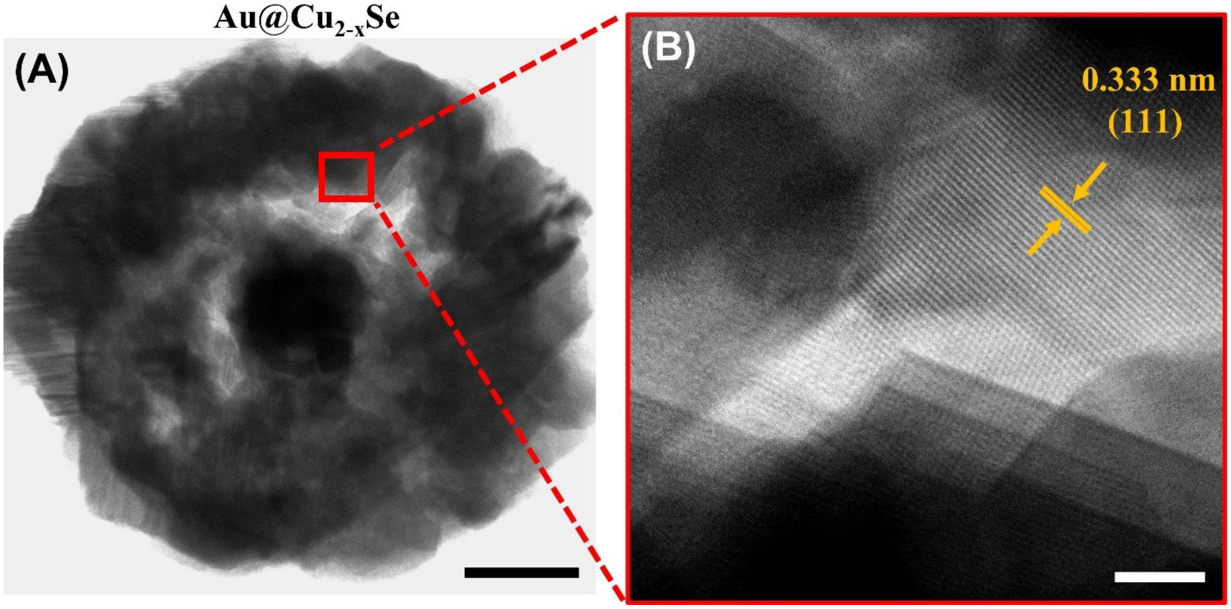

Fig. S3. (A) HRTEM image of an Au@$Cu_{2\text{-}x}Se$ nanoparticle with the denoted examined area. The scale bar is 50 nm. (B) HRTEM image of an Au@$Cu_{2\text{-}x}Se$ nanoparticle with indicated interplanar spacing corresponding to the lattice fringes of the (111) planes of the $Cu_{2\text{-}x}Se$ shell. The scale bar is 5 nm.

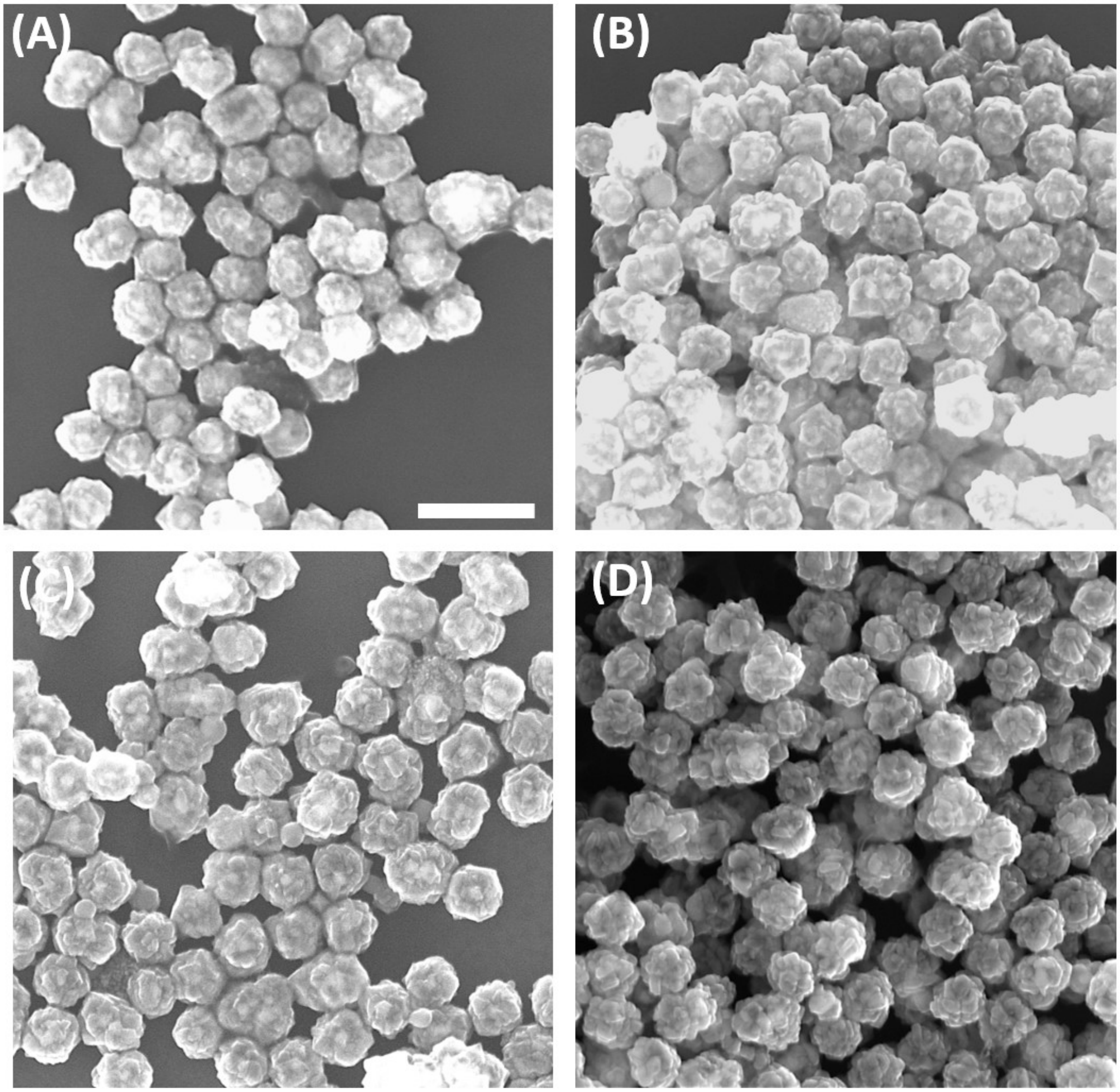

Fig. S4. SEM images of hollow Au@$Cu_{2-x}Se$ NPs obtained using (A) 10 μL, (B) 50 μL, (C) 100 μL, (D) 200 μL of 50 mM $(CH_3)_2NC(Se)NH_2$ aqueous solution. Scale bars correspond to 500 nm.

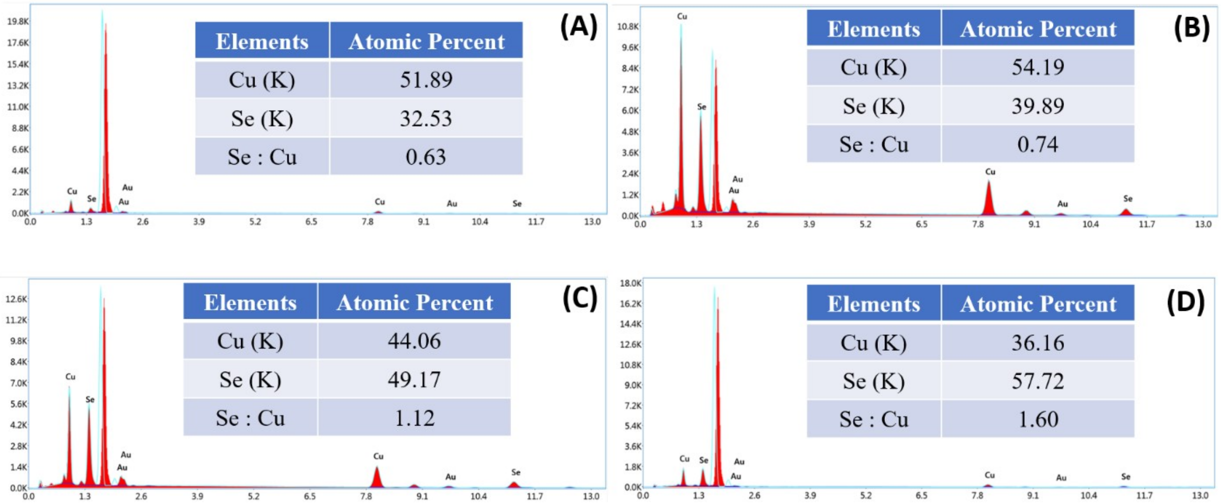

Fig. S5. The EDX spectra of hollow Au@Cu_{2-x}Se NPs obtained using (A) 10 μL, (B) 50 μL, (C) 100 μL, (D) 200 μL of 50 mM $(CH_3)_2NC(Se)NH_2$ aqueous solution.

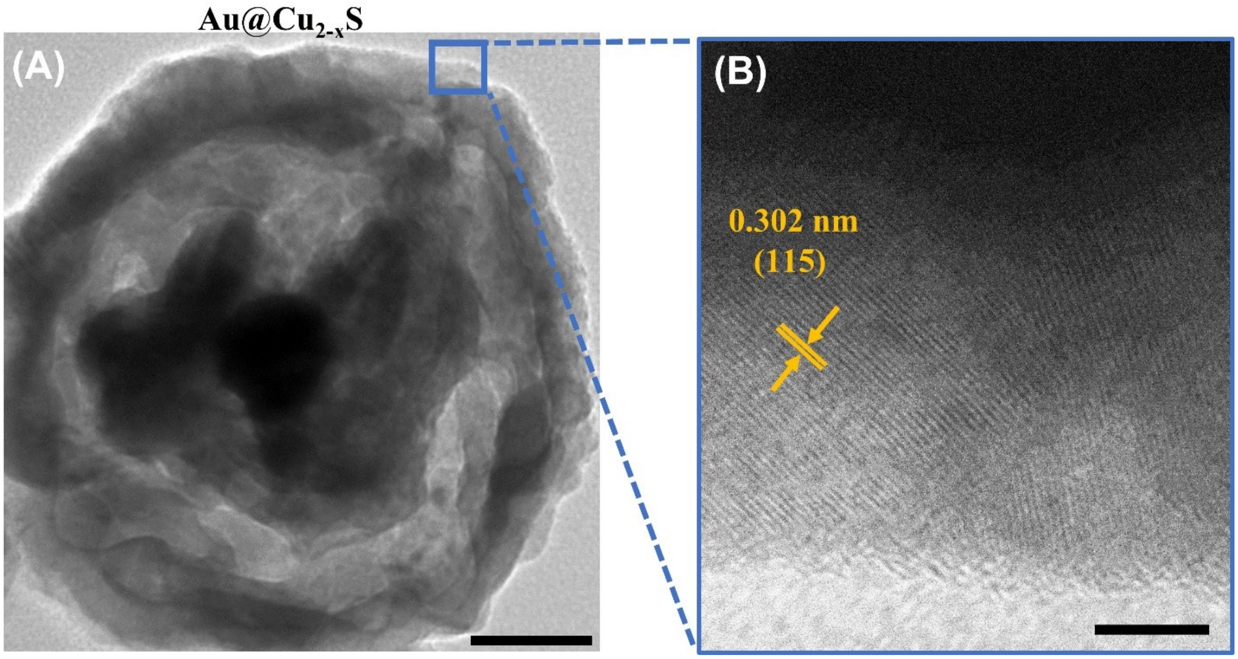

Fig. S6. (A) HRTEM image of an Au@Cu_{2-x}S nanoparticle with the denoted examined area. The scale bar is 50 nm. (B) HRTEM image of an Au@Cu_{2-x}S nanoparticle with indicated interplanar spacing corresponding to the lattice fringes of the (115) planes of the Cu_{2-x}S shell. The scale bar is 5 nm.

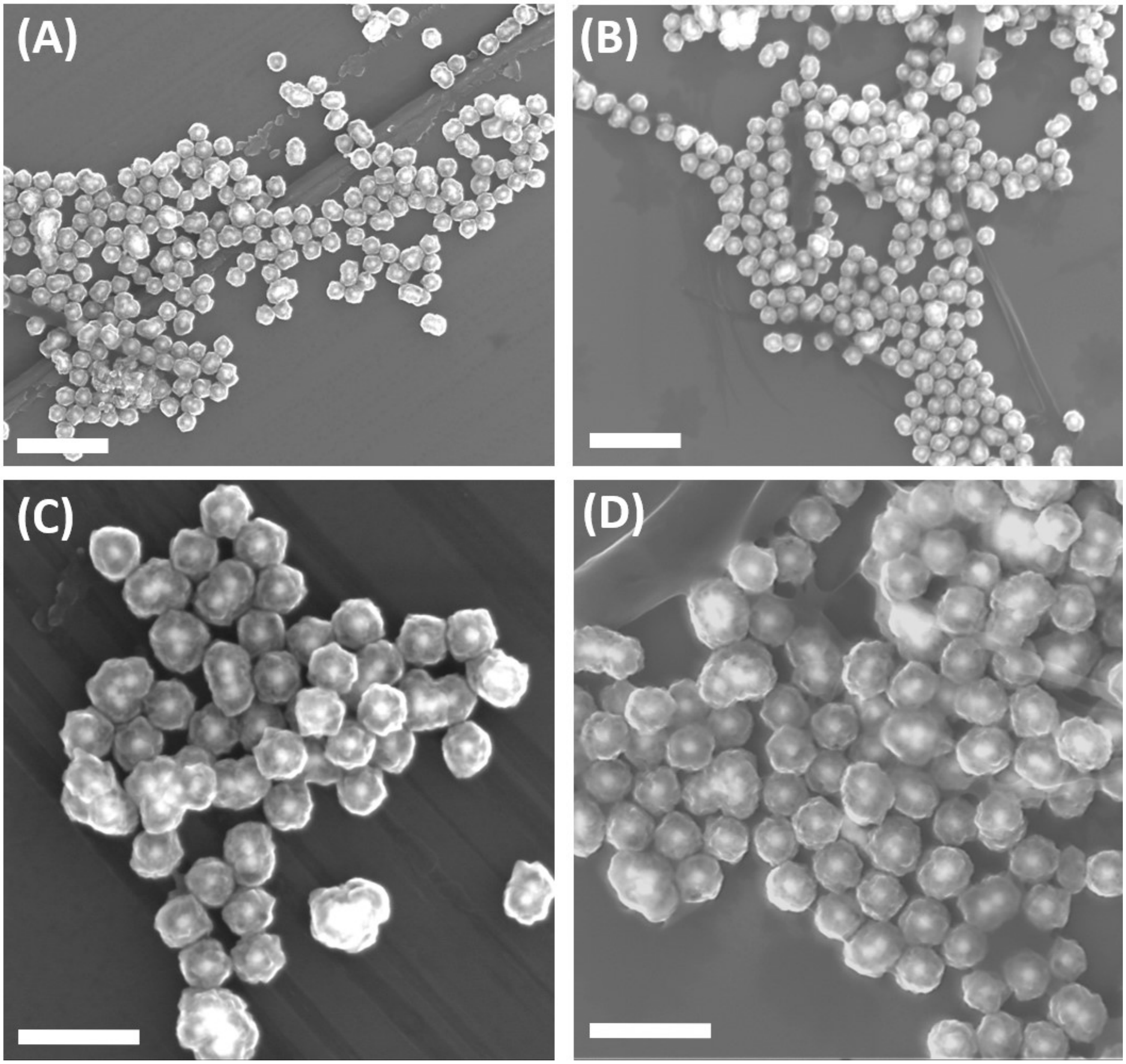

Fig. S7. SEM images of hollow Au@$Cu_{2-x}S$ NPs at high and low magnifications obtained using (A) 10 μL, (B) 200 μL of 50 mM $CH_3C(S)NH_2$, scale bars correspond to 1 μm; and (C) 10 μL, (D) 200 μL of 50 mM $CH_3C(S)NH_2$, scale bars correspond to 500 nm.

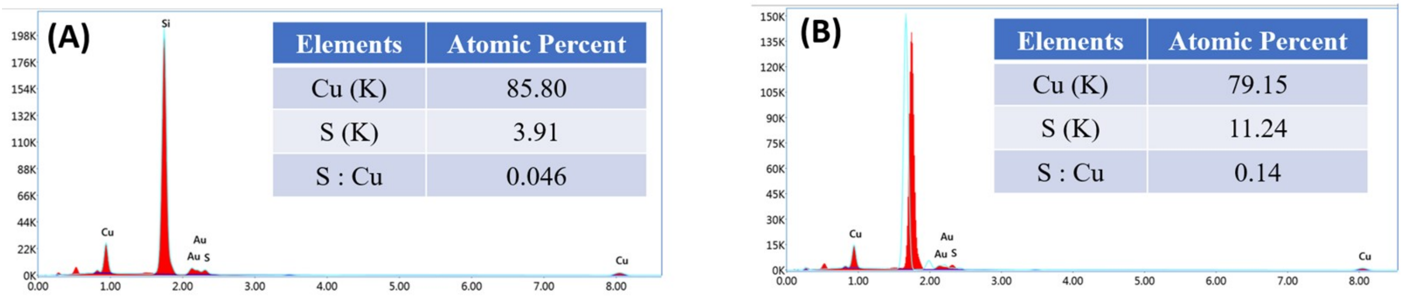

Fig. S8. The EDX spectra of hollow Au@$Cu_{2-x}S$ NPs obtained using (A) 10 µL, (B) 200 µL of 50 mM $CH_3C(S)NH_2$ aqueous solution.

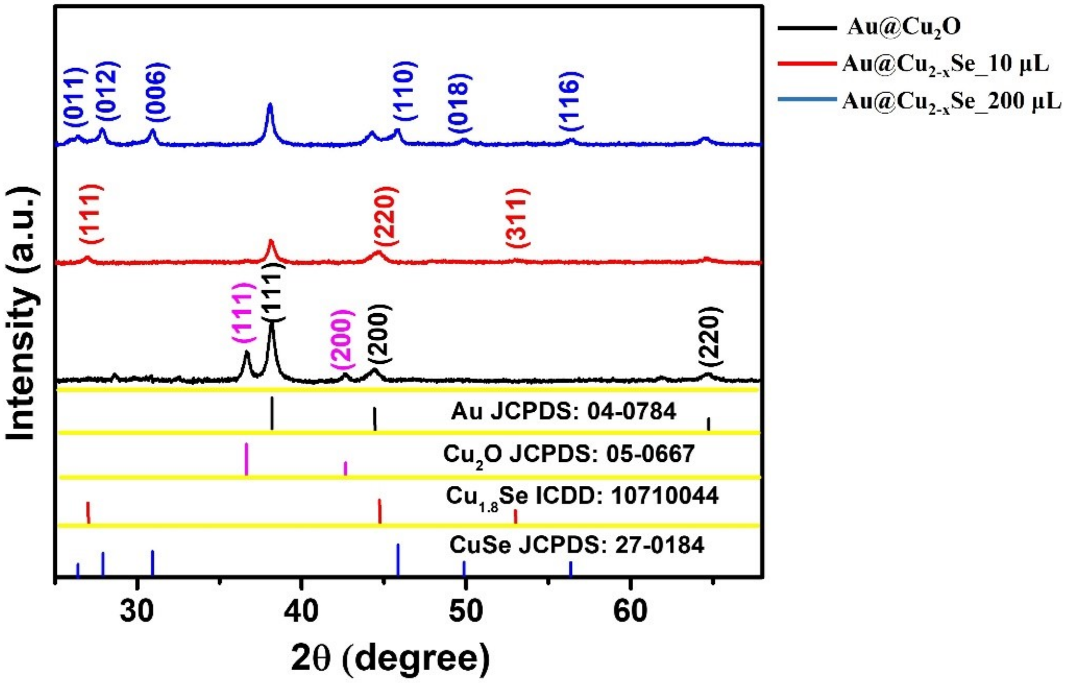

Fig. S9. XRD diffractograms of Au@Cu_2O NPs, hollow Au@$Cu_{2-x}Se$ NPs obtained using 10 µL and 200 µL of 50 mM $(CH_3)_2NC(Se)NH_2$ aqueous solution.

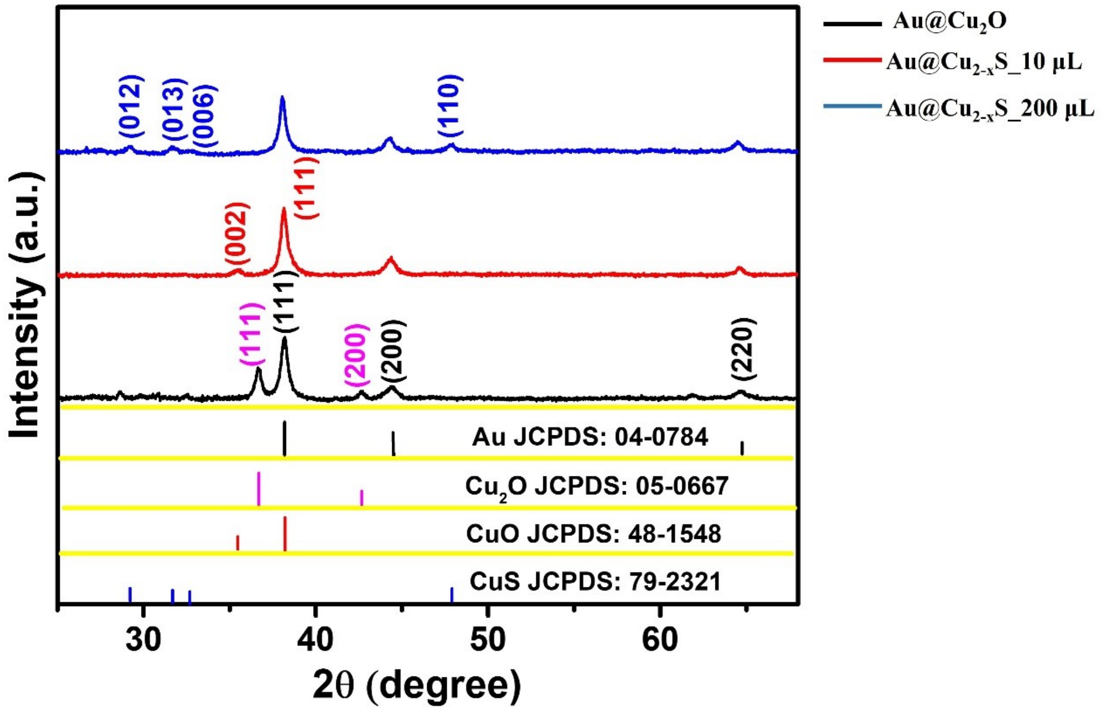

Fig. S10. XRD diffractograms of $Au@Cu_2O$ NPs, hollow $Au@Cu_{2-x}S$ NPs obtained using 10 µL, and 200 µL of 50 mM $CH_3C(S)NH_2$ aqueous solution.
